# Quality of clinical studies present in the package inserts of coagulation factors used in the treatment of hemophilia

**DOI:** 10.31744/einstein_journal/2022AO6859

**Published:** 2022-05-02

**Authors:** Yasmin Gonçalves Araújo, João Pedro Vasconcelos Paolinelli, Janaina Souza Dias Pichitelli, Danyelle Romana Alves Rios, Nayara Ragi Baldoni, André Oliveira Baldoni

**Affiliations:** 1 Universidade Federal de São João del Rei Divinópolis MG Brazil Universidade Federal de São João del Rei, Divinópolis, MG, Brazil.; 2 Universidade de Itaúna Itaúna MG Brazil Universidade de Itaúna, Itaúna, MG, Brazil.

**Keywords:** Hemophilia A, Hemophilia B, Medicine package inserts, Bias, Evidence-based practice

## Abstract

**Objective:**

To identify and analyze the quality of scientific evidence from clinical efficacy studies present in the package inserts of coagulation factors, used in the treatment of hemophilia A and B.

**Methods:**

Documentary study developed in two stages. The first stage consisted of identifying the medicine packages inserts electronically registered in the Brazilian Health Regulatory Agency, and analyzing the availability of the bibliographic references cited therein. This analysis was conducted in the PubMed, SciELO, Google Scholar, and Web of Science databases. The second step was the analysis of the methodological quality of the efficacy studies. Two trained researchers used the Cochrane Collaboration Risk of Bias version 5.1.0 tools for methodological quality analysis, and Review Manager 5.4 software to generate the risk of bias graph.

**Results:**

Of the 17 medicines listed, 7 had referenced package inserts. Of these, 10 studies were eligible for analysis of methodological quality. More than half of the analyzed studies did not control for selection, performance, and detection bias. A total of 100% controlled attrition and reporting biases, and 50% had a high risk of conflict of interest.

**Conclusion:**

The biases present are significant and may have influenced the overestimation of the effects of the outcomes of each of the studies.

## INTRODUCTION

Hemophilia is a disease characterized by qualitative or quantitative deficiencies of coagulation factors, with factor VIII (hemophilia A) and IX (hemophilia B) deficiencies being the most common.^(1)^ Patients with hemophilia manifest in the short or long term, hemorrhages into joints, muscles, or internal organs. According to the World Federation of Hemophilia (WFH), Brazil has the third largest population of patients with hemophilia in the world, with about 11,856 records.^(2,3)^

The treatment of hemophilia is based on replacement of the deficient factor to obtain adequate hemostasis.^(4)^ Treatment can be on demand (infusion of deficient coagulation factor concentrate during bleeding episodes) or prophylactic (regular infusion of factor concentrate at home). This prophylactic option was a great advance in the treatment of hemophilia, and its principles are the speed of infusion of the deficient factor, pain relief, reduction of hemophilic arthropathy, better clinical results and patients’ quality of life.^(5)^

For the treatment of hemophilia, factors of plasmatic origin and those of recombinant origin can be used. Factors of plasma origin are derived from human plasma and are currently considered safe due to purification techniques and viral inactivation. Concentrates of recombinant origin are highly purified, and made using molecular biology technology.^(5)^ One of the complications of hemophilia treatment is that some patients produce antibodies that inhibit the factors used in the treatment (*e.g*. anti-FVIII or anti-FIX). For these patients, additional therapy is required to overcome the need for factors VIII and IX, generating thrombin through other mechanisms. Some of the options are desmopressin, increase in the usual dose of coagulation factors, recombinant activated factor VII, and complex concentrate activated prothrombin.^(4,6)^

Coagulation factors are expensive pharmaceutical products. In Brazil they are acquired directly by the Ministry of Health, and in 2019 alone, the federal government made available BRL 1.3 billion for the purchase of medicines for the treatment of hereditary hemorrhagic diseases for the Public Health System (SUS - *Sistema Único de Saúde*).^(7)^The costs of a medicine depend on several factors, such as the technology used in the development and production process, as well as its proven efficacy and magnitude of benefits found in phase III clinical trials. Thus, through the evaluation of all documentation made available by pharmaceutical companies to request a medicine registration, regulatory agencies assess whether this new product is effective and safe, and whether the cost-benefit ratio is positive, as it is necessary to rationalize the health system resources.^(8,9)^

After the medicine approval and registration, the industry is still responsible for preparing the package inserts. These are informational materials for patients and health professionals, with easy and quick access, being considered one of the most important sources of information about medications after medical prescription, and for this reason they must be reviewed and inspected by the regulatory agency.^(9-12)^

The gold standard design for measuring intervention effects is the Randomized Clinical Trial, since this research design has strict methodological criteria, and parameters that allow controlling the influence of various factors on the investigated outcome.^(13)^ However, it is important to highlight that non-randomized, uncontrolled clinical studies, with no blinding, and other systematic errors have increased and are being used as a technical scientific basis for the approval of new treatments.^(14)^ These methodological flaws, which are called biases, may overestimate the benefits of the tested intervention, which can compromise the reliable analysis of the results and give the industries margins to increase the costs of these products.^(15,16)^

Given the budget constraint of the Ministry of Health of Brazil, combined with the high cost of coagulation factors used in the treatment of hemophilia, it is urgent to conduct research for assessing the quality of scientific evidence of phase III clinical studies available in the package inserts of these medicines, as there are many studies in which the scientific bias has been masked by economic interests.^(17,18)^

## OBJECTIVE

To identify and analyze the quality of scientific evidence from clinical efficacy studies present in the package inserts of medicines containing coagulation factors, used in the treatment of hemophilia A and B.

## METHODS

This is a documental study developed in two stages:

### Identification of the package inserts for health professionals and analysis of the availability of bibliographic references

To identify the references contained in the package inserts of coagulation factors available for distribution by the Ministry of Health, the inserts electronically registered in the Brazilian Health Regulatory Agency (ANVISA - *Agência Nacional de Vigilância Sanitária*) were consulted on June 20^th^, 2019 (http://portal.anvisa.gov.br/bulario-eletronico1). Table 1 shows the commercial presentations on the ANVISA website up to the consultation date.

**Table 1:** Coagulation factors distributed by the Ministry of Health for the treatment of hemophilia

Class	Commercial name
Factor VIII of plasma origin	8Y^®^; Advate^®^; Beriate P^®^; Biostate^®^; Fhandi^®^; Hemofil M^®^; Immunate^®^; Kogenate^®^; Octavi SDOptimum^®^; Optivate^®^
Factor VIII of recombinant origin	Hemo R8^®^
Factor VII of recombinant origin	Novoseven^®^
Factor IX of plasma origin	Factor IX Grifols^®^; Immunine^®^; Octanine F^®^; Replenine VF^®^
Partially activated prothrombin complex (factors II, IX, X, VII and factor VIII coagulant antigen)	Feiba^®^

The references described in the item “efficacy results” were analyzed in the package inserts for these medications. After identifying the references, the availability of full articles was analyzed in the PubMed, SciELO, Google Scholar, and Web of Science databases. Articles whose full texts were not available were requested from the industries that wrote the package inserts.

The articles found were categorized according to the methodological design used. Subsequently, all studies categorized as intervention studies were selected to identify their pre-clinical or clinical phase (phase I, II, III or IV).

### Analysis of the methodological quality of clinical studies contained in the package inserts

For this stage, only phase III clinical studies were eligible for analysis. All those intervention studies in humans, whose phase was not described in the article, but which aimed to evaluate the efficacy of the products, were also included. Pre-clinical studies, phase I, II and IV clinical studies, review studies, and observational studies were excluded from the analysis.

Individual analysis of the methodological quality of eligible clinical trials was performed using the Cochrane Collaboration 5.1.0 risk and bias tool. This tool consists of seven domains: - random sequence generation; - allocation concealment; - blinding of participants and professionals; - blinding of outcome evaluators; - incomplete outcomes; - selective outcome reporting; and - other sources of bias - for this domain, the conflict of interest in the analyzed studies was evaluated.^(19,20)^

Two trained researchers performed the analysis and subsequently met to discuss the necessary adjustments as for possible disagreements. At the end of the analysis, a graph with the risk of bias was generated using Review Manager 5.4 software. The methodological components of the studies were categorized into low risk of bias, high risk of bias, or unclear risk of bias.^(20)^

Thus, the biases evaluated by the tool are: - selection bias (domains 1 and 2): systematic differences between the baseline characteristics of the compared groups; - performance bias (domain 3): systematic differences between groups in the care provided or in exposure to factors other than the interventions of interest; - detection bias (domain 4): systematic differences between groups during outcome analysis; - attrition bias (domain 5): systematic differences between groups in data loss and/or participants; - reporting bias (domain 6): systematic differences between reported and unreported outcomes; - other biases - conflict of interest bias - (domain 7): presence of a conflict of interest of any origin.^(19,20)^

## RESULTS

**Figure 1:**
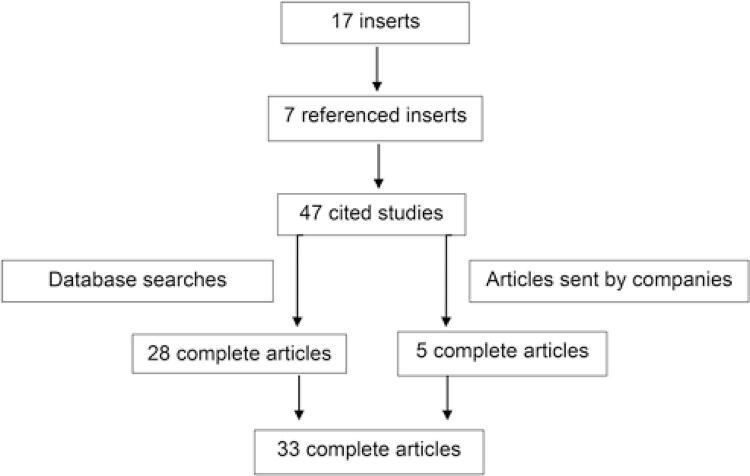
Availability of references cited in the package inserts of coagulation factors used in the treatment of hemophilia

After analyzing the package inserts of the 17 listed medicines, only 7 contained references. Of these, 47 referenced studies were identified. After searching the databases, 28 studies were accessed. Some companies responded and sent 5 studies requested by email (Figure 1). At the end of the searches, 33 complete articles were obtained, which were categorized according to their design (Table 2).

**Table 2:** Categorization by methodological design of the studies present in the package inserts of each medicine

Medicine	Class	Total	Observational	Review	Intervention study
Advate^®^	Factor VIII of plasma origin	5	3	--	2
Biostate^®^	Factor VIII of plasma origin	2	2	--	--
Feiba^®^	Partially activated prothrombin complex	4	2	--	2
Hemo 8r^®^	Factor VIII of recombinant origin	1	--	1	--
Hemofil^®^	Factor VIII of plasma origin	3	--	3	--
Immunate^®^	Factor VIII of plasma origin	1	--	--	1
Novoseven^®^	Factor VII of recombinant origin	7	1	1	5
Optivate^®^	Factor VIII of plasma origin	2	2	--	--
Replenine VF^®^	Factor IX of recombinant origin	8	7	--	1
Total		33	17	5	11

Clinical studies described as phase III and all those that aimed to assess the efficacy and safety of medications were selected for the second stage of the present study. It is noteworthy that the study by Ewenstein et al.^(21)^ mentioned in the package insert of the Replenine VF^®^ product, was not eligible for the second stage of the study, as its objective was only to compare pharmacokinetics (Table 3).

**Table 3:** Profile of intervention studies present in the package inserts of coagulation factors used in the treatment of hemophilia

Medicine	Authors	Phase	Objective	Control Group
Advate^®^	Tarantino et al.,^(22)^	ND	Bioequivalence, pharmacokinetic, safety and efficacy	No
Advate^®^	Négrier et al.,^(23)^	ND	Efficacy and safety	No
Feiba^®^	Astermark et al.,^(24)^	ND	Efficacy	Yes
Feiba^®^	Sjamsoedin et al.,^(25)^	ND	Efficacy	Yes
Immunate^®^	Nemes et al.,^(26)^	III	Efficacy and safety	No
Novoseven^®^	Key et al.,^(27)^	III	Efficacy and safety	No
Novoseven^®^	Scharrer et al.,^(28)^	ND	Efficacy and safety	No
Novoseven^®^	Shapiro et al.,^(29)^	ND	Efficacy and safety	Yes
Novoseven^®^	Kavakli et al.,^(30)^	ND	Efficacy and safety	Yes
Novoseven^®^	Young et al.,^(31)^	ND	Efficacy and safety	Yes
Replenine VF^®^	Ewenstein et al.,^(21)^	ND	Pharmacokinetics	No

ND: no description.

It is important to highlight that of the 11 intervention studies analyzed, 10 were eligible for the analysis of methodological quality. Of these, 50% (n=5) conducted the study without a Control Group.

**Figure 2:**
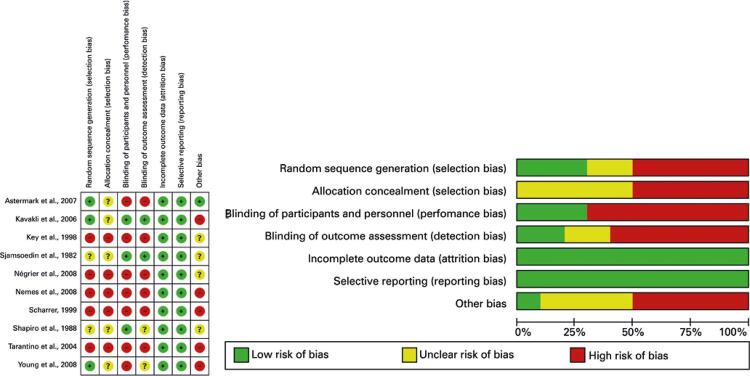
Risk of bias summary: analysis of the authors judgments on each risk of bias item for each included study

More than 50% of the analyzed studies did not control for the selection, performance, and detection bias, as they present a high risk of bias or uncertainties as for methodological quality. When analyzing by domain generation of random sequence (domain 1) and concealment of allocation (domain 2), 50% of the studies presented a high risk of bias for both domains. Regarding the blinding of participants and professionals (domain 3) and blinding of outcome evaluators (domain 4), a high risk of bias in 70% and 60% of the studies, respectively was found. As for the incomplete outcomes (domain 5) and selective outcome reporting (domain 6) domains, 100% of the studies had a low risk of bias. Finally, for domain 7 conflict of interest, 50% of the studies presented a high risk of bias (Figure 2).

## DISCUSSION

The results of this study showed, in general, that clinical studies aimed at evaluating efficacy and safety referred to in the package inserts of medicines containing coagulation factors for the treatment of hemophilia, have methodological flaws. Among the domains evaluated, those with a high risk of bias in most studies were the blinding domains. Furthermore, in three domains, 50% of the studies were classified as high risk of bias (random sequence generation, allocation concealment, and conflict of interest). These weaknesses can overestimate efficacy effects and compromise the reliable analysis of results.

A large portion of the analyzed studies were not able to control the selection bias. This finding can be explained by the fact that 50% of the analyzed studies did not have a Control Group, and when they did, did not report on the performance and/or methods of randomization and allocation. These two steps are essential in a clinical trial to alleviate selection bias, thus providing baseline similarity, that is, the generation of similar groups in terms of their characteristics and baseline known and unknown risk factors. Similar groups in clinical trials contribute to the effect estimates obtained in the studies, being closer to the true result.^(32,33)^ In contrast, it is important to recognize that due to the fact that hemophilia is a low-prevalence disease that directly impacts on patients’ health and quality of life, the use of Control Groups using pure placebo is limited. However, in an attempt to minimize biases, increase statistical power, and at the same time not violate ethical principles in human research, small groups could have been cross-randomized into intervention and usual care groups.^(3,34,35)^ Therefore, it is important to be cautious when interpreting the results of the studies analyzed here, since most of them did not control for selection bias.

Most studies analyzed did not control for performance bias and detection bias. It is important to emphasize that blinding is only possible in studies with Control Groups, as the results of outcomes assessed in single-arm studies are only those of the applied intervention.^(36)^ Of the five studies that used Control Groups, only two were with blinded outcome evaluators, and three with blinded participants and professionals. According to Buehler et al.,^(34)^ and Kamper^(37)^ it is not always possible to apply blinding, possibly because it is difficult to perform blinding in studies that assess the efficacy and safety of coagulation factors for the treatment of hemophilia, as they are injectable preparations. On the other hand, some alternatives could have been used to overcome this barrier, like those used in the study by Sjamsoedin et al.,^(25)^ such as coding interventions and using dark colored bottles and opaque syringes. The absence of blinding in clinical trials directly implies the estimation of the effect of the evaluated outcome. Schulz et al.^(36)^ evaluated the impact of methodological quality on the effect estimate and showed that studies that were not double-blind produced effect estimates 17% higher than those that were double-blind. In this context, it is clear that once again, the results reported in the studies contained in the coagulation factors package inserts may not be as encouraging, as the reported effect estimates may also have been influenced by the performance and detection bias.

Furthermore, the present study showed that all the analyzed studies controlled the attrition bias. A well-conducted study depends on adequate patient follow-up and the preservation of collected data. Data loss can affect the veracity of the effects of each outcome, and unexplained loss of patients can hide a possible failure in the protocol or even a safety issue in the treatment.^(38)^ Most of the studies analyzed here performed adequate follow-up and all losses and exclusions were duly justified. It is noteworthy, therefore, that the attrition bias was controlled and, although the size of the treatment effect is questionable due to the presence of selection, performance, and detection biases, it can be judged with more confidence, that the evaluated treatments were safe for most of the analyzed studies.

Moreover, a positive point of the analyzed studies is that all of them controlled the reporting bias, as they presented a low risk of bias in the selective outcome report domain. Controlling reporting bias is essential so that there are no deviations in study protocols, such as the inversion of clinical outcomes (primary) by substitute (secondary) outcomes. This can occur intentionally, when, *e.g*., the authors assess multiple outcomes but report only those that had positive effects, leading to erroneous conclusions as for the efficacy of the studied intervention.^(11,39)^ Therefore, it is notable that the authors reported the proposed primary and secondary outcomes, thus evidencing results that are of interest to the perspectives of health professionals and patients.

Furthermore, this research evidenced that half of the studies showed a conflict of interest for financing from companies interested in the approval of the medicine. Palma et al.,^(40)^ bring in their study a reflection that the interest of the pharmaceutical industry in research, production, dissemination, and commercialization of medicines is a reality all over the world. A study conducted by Santos et al.^(41)^ explained that the conflict of interest was present in most of the studies analyzed and that funding by the pharmaceutical industry was associated with conclusions favorable to the treatment being tested. That said, it is crucial to critically analyze the results of clinical trials where industry funding is available, as this study design is largely dependent on rigorous methods to control bias, and any deviation, whether intentional or not, can influence in an increase in the estimated effect of the evaluated outcome.

Before requesting the registration of a drug, the pharmaceutical industry must comply with current legislation and present to regulatory agencies documentation containing pre-clinical and clinical studies to prove efficacy and safety, in addition to administrative technical issues, such as certificates of good manufacturing practices. It is also responsibility of the industry to prepare the package insert.^(9)^ The Collegiate Board of Directors Resolution (RDC) # 47 of 2009^(11)^ regulates, standardizes, and assists in the preparation and updating of information on medicine package inserts in the country. This resolution provides that the reference, generic and similar package inserts, intended to health professionals, must describe the results of the studies used to prove the efficacy and registration of the drug, as well as bibliographical references of the cited studies.

This study brings worrying data as for the quality of technical information present in the package inserts for coagulation factors, showing that 10 of the 17 package inserts selected for health professionals do not refer to the described efficacy studies. The RDC # 47 of 2009,^(11)^ which standardizes and assists in the preparation and updating of information on medicine package inserts for patients and health professionals, establishes that the described efficacy results must cite the bibliographic references used. Marques et al.^(42)^ reinforce that in order not to negatively compromise the evidence-based clinical practice, a greater commitment of the industries in the adaptation to the current legislation for the production of package inserts, and greater rigor in the approval and inspection of these by the sanitary regulatory agencies is necessary. In this context, ANVISA needs to review and update the package inserts of products containing coagulation factors for the treatment of hemophilia in Brazil, so that health professionals have available quality and updated information, to enable them to seek the primary sources of the cited scientific evidence, allowing them to make a critical analysis of each of the studies used for the approval and use of the medicine.

This study has some limitations, such as the impossibility of accessing articles that are not freely accessible. However, to the best of our knowledge, this study brought important reflections on the quality of scientific evidence used to prove the efficacy and registration of medicines, technical quality in the production, updating and inspection of package inserts, and the possible influence of the pharmaceutical industry in this context. Therefore, this should be a topic for discussion and investigation by all actors involved in this process, including researchers, the pharmaceutical industry, the regulatory agency, health managers, and professionals.

Finally, it is important to emphasize that the authors are aware that the medicines approved for the treatment of hemophilia were a revolution in improving the patients’ quality of life and health, and for this reason the objective here was not to question innovative studies and proven effective pharmacological treatments, but rather to identify and explain the methodological gaps existing in the studies that support the approval for the use and commercialization of medicines.

## CONCLUSION

The present study showed that the quality of scientific evidence available on the efficacy of medicines containing coagulation factors used in the treatment of hemophilia needs to be carefully analyzed and interpreted, as the biases present are significant from a methodological and clinical point of view. The results of this study suggest that the high frequencies of bias in these studies may have influenced the overestimation of outcome results in each of them. Furthermore, the available package inserts should be revised to adapt to current standards, with the aim of enhancing and improving the information intended for health professionals.

## References

[1] Peyvandi F, Duga S, Akhavan S, Mannucci PM. Rare coagulation deficiencies. Haemophilia. 2002;8(3):308-21. Review. 10.1046/j.1365-2516.2002.00633.x 12010428

[2] Ferreira AA, Leite IC, Bustamante-Teixeira MT, Guerra MR. Hemophilia A in Brazil- epidemiology and treatment developments. J Blood Med. 2014;5:175-84. Review. 10.2147/JBM.S68234 PMC4183441 25288890

[3] Brasil. Ministério da Saúde. Secretaria de Atenção à Saúde. Departamento de Atenção Especializada e Temática. Perfil das coagulopatias hereditárias: 2016. Brasília (DF): Ministério da Saúde; 2018 [citado 2021 Fev 18]. Disponível em: https://bvsms.saude.gov.br/bvs/publicacoes/perfil_coagulopatias_ hereditarias_2016.pdf

[4] Peyvandi F, Garagiola I, Young G. The past and future of haemophilia: diagnosis, treatments, and its complications. Lancet. 2016;388(10040):187-97. Review. 10.1016/S0140-6736(15)01123-X 26897598

[5] Brasil. Ministério da Saúde. Secretaria de Atenção à Saúde. Departamento de Atenção Especializada e Temática. Manual de hemofilia. Brasília (DF): Ministério da Saúde; 2015 [citado 2021 Fev 18]. Disponível em: https://bvsms.saude.gov.br/bvs/publicacoes/manual_hemofilia_2ed.pdf

[6] Brasil. Ministério da Saúde. Secretaria de Atenção à Saúde. Departamento de Atenção Especializada. Hemofilia congênita e inibidor: manual de diagnóstico e tratamento de eventos hemorrágicos. Brasília (DF): Ministério da Saúde; 2009 [Série A. Normas e Manuais Técnicos] [citado 2021 Fev 18]. Disponível em: https://bvsms.saude.gov.br/bvs/publicacoes/hemofilia_congenita_inibidor_diagnostico_tratamento.pdf

[7] Brasil. Ministério da Saúde. Governo do Brasil. Ministério da Saúde garante R$ 1,3 bilhão para tratamento de hemofílicos. Brasília (DF): Ministério da Saúde; 2019 [citado 2021 Mar 25]. Disponível em: https://www.gov.br/pt-br/noticias/saude-e-vigilancia-sanitaria/2019/04/ministerio-da-saude-garanter-1-3-bilhao-para-tratamento-de-hemofilicos

[8] Wallerstedt SM, Hoffmann M. Evaluating beneficial drug effects in a non-interventional setting: a review of effectiveness studies based on Swedish Prescribed Drug Register data. Br J Clin Pharmacol. 2017;83(6):1309-18. Review. 10.1111/bcp.13206 PMC5427236 27928842

[9] Brasil. Câmara dos Deputados. Centro de Documentação e Informação. Lei n. 6.360, de 23 de Setembro de 1976. Dispõe sobre a vigilância sanitária a que ficam sujeitos os medicamentos, as drogas, os insumos farmacêuticos e correlatos, cosméticos, saneantes e outros produtos, e dá providências. Brasília (DF): Diário Oficial da União; 1976 [citado 2021 Mar 25]. Disponível em: https://www2.camara.leg.br/legin/fed/lei/1970-1979/lei-6360-23-setembro-1976-357079-normaatualizada-pl.html

[10] Caldeira TR, Neves ER, Perini E. Evolução histórica das bulas de medicamentos no Brasil. Cad Saude Publica. 2008;24(4):737-43. Review. 10.1590/s0102-311x2008000400003 18392350

[11] Brasil. Resolução RDC nº 47, de 8 de setembro de 2009. Estabelece regras para elaboração, harmonização, atualização, publicação e disponibilização de bulas de medicamentos para pacientes e para profissionais de saúde. Brasília (DF): Diário Oficial da União, 19 jan, 2010 [citado 2021 Mar 26]. Disponível em: https://pesquisa.in.gov.br/imprensa/jsp/visualiza/index.jsp?data=19/01/2010&jornal=1&pagina=36&totalArquivos=72

[12] da Silva T, Dal-Pizzol F, Bello CM, Mengue SS, Schenkel EP. Bulas de medicamentos e a informação adequada ao paciente. Rev Saude Publica. 2000;34(2):184-9. 10.1590/s0034-89102000000200013 10881155

[13] Escosteguy, CC. Estudos de Intervenção. In: Medronho RA. Epidemiologia. 2a ed. São Paulo: Atheneu; 2008. p. 251-63.

[14] Wallerstedt S. Nya läkemedel kan nomineras för introduktionsfinansiering - Prioriteringen bygger på medicinsk och vetenskaplig värdering – goda erfarenheter från process i Västra Götalandsregionen. Lakartidningen. 2016; 113:D43D. 27779725

[15] Borges M. Ensaios Clínicos em Medicamentos. Revista Portuguesa de Cirurgia. 2013:57-63.

[16] Thorat T, Neumann PJ, Chambers JD. Hemophilia burden of disease: a systematic review of the cost-utility literature for hemophilia. J Manag Care Spec Pharm. 2018;24(7):632-42. Review. 10.18553/jmcp.2018.24.7.632 PMC10397835 29952709

[17] Kunin CM. Clinical Investigators and the pharmaceutical industry. Ann Intern Med. 1978;89(5 Pt 2 Suppl):842-5. 10.7326/0003-4819-89-5-842 717966

[18] Angell M. Is academic medicine for sale? N Engl J Med. 2000;342(20):1516-8. 10.1056/NEJM200005183422009 10816191

[19] Carvalho AP, Silva V, Grande AJ. Avaliação do risco de viés de ensaios clínicos randomizados pela ferramenta da colaboração Cochrane. Diagn Tratamento. 2013;18(1):38-44.

[20] Cochrane Training. Cochrane Handbook for Systematic Reviews of Interventions. London: Cochrane; 2021 [cited 2021 Mar 6]. Available from: https://training.cochrane.org/handbook

[21] Ewenstein BM, Joist JH, Shapiro AD, Hofstra TC, Leissinger CA, Seremetis SV, Broder M, Mueller-Velten G, Schwartz BA; Mononine Comparison Study Group. Pharmacokinetic analysis of plasma-derived and recombinant F IX concentrates in previously treated patients with moderate or severe hemophilia B. Transfusion. 2002;42(2):190-7. 10.1046/j.1537-2995.2002.00039.x 11896334

[22] Tarantino MD, Collins PW, Hay CR, Shapiro AD, Gruppo RA, Berntorp E, Bray GL, Tonetta SA, Schroth PC, Retzios AD, Rogy SS, Sensel MG, Ewenstein BM; RAHF-PFM Clinical Study Group. Clinical evaluation of an advanced category antihaemophilic factor prepared using a plasma/albumin-free method: Pharmacokinetics, efficacy, and safety in previously treated patients with haemophilia A. Haemophilia. 2004;10(5):428-37. 10.1111/j.1365-2516.2004.00932.x 15357767

[23] Négrier C, Shapiro A, Berntorp E, Pabinger I, Tarantino M, Retzios A, et al. Surgical evaluation of a recombinant factor VIII prepared using a plasma/albumin-free method: efficacy and safety of advate in previously treated patients. Thromb Haemost. 2008;100(2):217-23. 18690340

[24] Astermark J, Donfield SM, DiMichele DM, Gringeri A, Gilbert SA, Waters J, Berntorp E; FENOC Study Group. A randomized comparison of bypassing agents in hemophilia complicated by an inhibitor: the FEIBA NovoSeven Comparative (FENOC) Study. Blood. 2007;109(2):546-51. 10.1182/blood-2006-04-017988 16990605

[25] Sjamsoedin LJ, Heijnen L, Mauser-Bunschoten EP, van Geijlswijk JL, van Houwelingen H, van Asten P, et al. The effect of activated prothrombin-complex concentrate (FEIBA) on joint and muscle bleeding in patients with haemophilia A and antibodies to factor VIII: a double blind clinical trial. N Engl J Med. 1981;305(13):717-21. 10.1056/NEJM198109243051301 6790990

[26] Nemes L, Lissitchkov T, Dobaczewski G, Klukowska A, Komrska V, Zimmermann R, et al. Pharmacokinetics, efficacy and safety of IMMUNATE^®^ Solvent/Detergent (IMMUNATE^®^ S/D) in previously treated patients with severe hemophilia A: results of a prospective, multicenter, open-label phase III study. Acta Haematol. 2008;119(2):89-97. 10.1159/000118628 18305381

[27] Key NS, Aledort LM, Beardsley D, Cooper HA, Davignon G, Ewenstein BM, et al. Home treatment of mild to moderate bleeding episodes using recombinant factor VIIa (Novoseven) in haemophiliacs with inhibitors. Thromb Haemost. 1998;80(6):912-8. 9869160

[28] Scharrer I. Recombinant factor VIIa for patients with inhibitors to factor VIII or IX or factor VII deficiency. Haemophilia. 1999;5(4):253-9. 10.1046/j.1365-2516.1999.00319.x 10469179

[29] Shapiro AD, Gilchrist GS, Hoots WK, Cooper HA, Gastineau DA. Prospective, randomised trial of two doses of rFVIIa (NovoSeven) in haemophilia patients with inhibitors undergoing surgery. Thromb Haemost. 1998;80(5):773-8. 9843170

[30] Kavakli K, Makris M, Zulfikar B, Erhardtsen E, Abrams ZS, Kenet G; NovoSeven trial (F7HAEM-1510) investigators. Home treatment of haemarthroses using a single dose regimen of recombinant activated factor VII in patients with haemophilia and inhibitors. A multi-centre, randomised, double-blind, cross-over trial. Thromb Haemost. 2006;95(4):600-5. 16601828

[31] Young G, Shafer FE, Rojas P, Seremetis S. Single 270 microg kg(-1) -dose rFVIIa vs. standard 90 microgkg(-1) -dose rFVIIa and APCC for home treatment of joint bleeds in haemophilia patients with inhibitors: a randomized comparison. Haemophilia. 2008;14(2):287-94. 10.1111/j.1365-2516.2007.01601.x 18081834

[32] Fregnani JH, Carvalho AL, Paranhos FR, Viana LS, Serrano SV, Cárcano F, et al. Eticidade do uso de placebo em pesquisa clínica: proposta de algoritmos decisórios. Rev Bioét. 2015;23(3):456-67.

[33] Fletcher RH, Fletcher SW. Epidemiologia Clínica:elementos essenciais. 4a ed. Porto Alegre: Artmed; 2006. p. 1-18.

[34] Buehler AM, Cavalcanti AB, Suzumura EA, Carballo MT, Berwanger O. How to assess intensive care randomized trials. Rev Bras Ter Intensiva. 2009; 21(2):219-25. 25303354

[35] Kamper SJ. Blinding: linking evidence to practice. J Orthop Sports Phys Ther. 2018;48(10):825-6. 10.2519/jospt.2018.0705 30270781

[36] Schulz KF, Chalmers I, Hayes RJ, Altman DG. Empirical evidence of bias: Dimensions of methodological quality associated with estimates of treatment effects in controlled trials. JAMA.1995;273(5):408-12. 10.1001/jama.273.5.408 7823387

[37] Kamper SJ. Randomization: linking evidence to practice. J Orthop Sports Phys Ther. 2018;48(9):730-1. 10.2519/jospt.2018.0704 30170525

[38] Froud R, Bjørkli T, Bright P, Rajendran D, Buchbinder R, Underwood M, et al. The effect of journal impact factor, reporting conflicts, and reporting funding sources, on standardized effect sizes in back pain trials: A systematic review and meta-regression. BMC Musculoskelet Disord. 2015;16:370. Review. 10.1186/s12891-015-0825-6 PMC4663726 26620449

[39] Wallerstedt SM, Wettermark B, Hoffmann M. The first decade with the swedish prescribed drug register - a systematic review of the output in the scientific literature. Basic Clin Pharmacol Toxicol. 2016;119(5):464-9. Review. 10.1111/bcpt.12613 27112967

[40] Palma A, Vilaça MM. Conflitos de interesse na pesquisa, produção e divulgação de medicamentos. Hist Cienc Saude - Manguinhos. 2012;19(3):919-32. 10.1590/s0104-59702012000300008 23070379

[41] Santos M, Silva DA, Paranhos FR. Conflito de interesses em ensaios clínicos iniciais envolvendo pacientes com neoplasia de pulmão. Rev Bioét. 2014;22(3):500-8.

[42] Marques LO, Vasconcelos RC, Baldoni AO, Pestana AC, Chequer FM. Cardiovascular drug labeling: Do they have information on necessary precautions for older people? Geriatr Gerontol Aging. 2020;14(3):196-202.

